# Effect of sevoflurane anaesthesia on nasal mask in endoscopic retrograde cholangiopancreatography: is it a preferred alternative?

**DOI:** 10.3906/sag-1910-60

**Published:** 2020-04-09

**Authors:** Bülent BALTACI, Hülya BAŞAR, Murat KEKİLLİ, Mert NAKİP, Fatih KARAAHMET, Mehmet ÇAKIRCA, Melis ENGİN, Meltem BEKTAŞ

**Affiliations:** 1 Department of Anesthesiology and Reanimation, Ankara Training and Research Hospital, Ankara Turkey; 2 Department of Gastroenterology, Gazi University, Hospital of Faculty of Medicine, Ankara Turkey; 3 Department of Gastroenterology, Ankara Training and Research Hospital, Ankara Turkey

**Keywords:** Sedation, endoscopic retrograde cholangiopancreatography, sevoflurane, nasal mask

## Abstract

**Background/aim:**

Endoscopic retrograde cholangiopancreatography (ERCP) often requires deep sedation. Propofol provides adequate sedation and amnesia at subhypnotic doses, but safe guarding the patient’s airway is important for preventing respiratory depression or u28ba events. This study compared sedation levels, operator satisfaction, intraoperative and recovery characteristics using sevoflurane with nasal mask and propofol in ERCP.

**Material and methods:**

Sixty-one patients underwent ERCP (Group I: propofol, n = 31; Group II, sevoflurane, n = 30), with sedation controlled by the Ramsay sedation scale (RSS). The patients’ demographic data, procedure length, overall drug dose, hemodynamic changes, duration of recovery and Aldrete scores during recovery were evaluated. In addition, satisfaction of the gastroenterologist was evaluated.

**Results:**

The mean sphincterotomy satisfaction scores were statistically significant (P= 0.043). The Aldrete scores and RSS of the groups were similar; there was a significant difference between groups at the beginning of the procedure regarding peripheric oxygen saturations and Group II’s saturation levels increased during sedation.

**Conclusion:**

In ERCP, propofol infusion provides shorter recovery duration and adequate sedation levels. Sevoflurane and oxygen with a nasal mask can be chosen to generate specific anaesthesia in patients, especially for strong airway support and safety treating hypoxemic patients.

## 1. Introduction

Endoscopic retrograde cholangiopancreatography (ERCP) is an invasive procedure for both diagnosis and treatment of pancreatic and biliary pathologies. The process requires a certain depth of sedation. The purpose of conscious sedation is to keep verbal communication while ensuring the patient unresponsive to painful stimuli [1]. Patients should be kept at least at the level of conscious sedation or slightly deeper. Sedation scoring can be used to achieve this; in our study, the Ramsay sedation scale (RSS) scoring was employed. 

General anaesthesia may be preferred, especially for patients with severe cardiac and respiratory problems. Under general anaesthesia, the patient’s airway is safer and hemodynamic instability can be controlled better. Nitrous oxide and nasal masks, as well as adequate sedation levels, are frequently applied in patients with paediatric dental problems [2]. Obstructive Sleep Apnoea Syndrome (OSAS) patients using bi-level positive airway pressure device (BIPAP) can also receive oxygen support with their devices in nonoperating room procedures [3].

ERCP is performed under conscious sedation using an intravenous (IV) anaesthetic drug. Propofol is a highly preferred IV agent for processes requiring sedation. It can be used in the form of slow infusions or boluses. Inadequate conscious sedation has been reported in 14% of ERCP patients [4]; an insufficient level of sedation represents an important factor in ERCP failure [5].

In this study, 2 different methods were used for sedation in ERCP cases. We could not find a publication in the literature on the administration of sevoflurane using a nasal mask. The aim of this study was to compare sedation levels, operator satisfaction and intraoperative and recovery characteristics using sevoflurane with a nasal mask and propofol in ERCP procedures.

## 2. Material and methods

This prospective observational study was performed in the Anaesthesiology and Reanimation Clinic and Gastroenterology Department of the Ankara Training and Research Hospital with the approval of the local ethics committee. ERCP procedures were performed in the gastroenterology endoscopy unit between January and March 2019 and evaluated prospectively. All the ERCP procedures in our hospital are performed by 2 gastroenterologists, with sedation administered by an anaesthesia specialist. The patients’ clinical data were screened for their suitability for inclusion in this study. Patients who had ERCP were involved in the study. Patients with a physical condition classification score of II–III who had fasted for at least 8 h and were undergoing ERCP for the first time were included in the study. Patients with severe heart failure and arrhythmia, cardiac pacemakers or allergies to drugs were excluded. 

**Figure 1:**
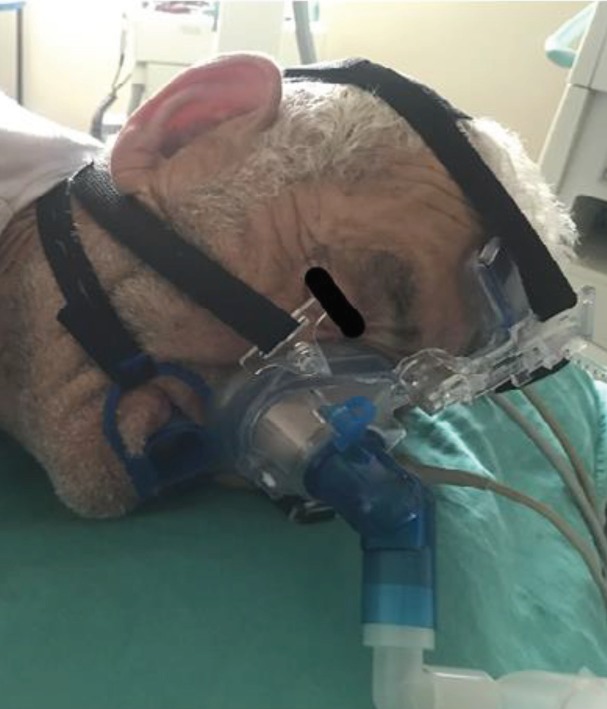
Nasal mask placed on face.

The bispectral index (BIS) or RSS monitoring is routinely used in our clinic to evaluate the depth of anaesthesia during ERCP. We preferred to use RSS in this study. The BIS probe could not be used because of the presence of the nasal mask’s forehead apparatus (Figure 1).

Each patient’s heart rate (HR), systolic arterial blood pressure (SAP), diastolic arterial blood pressure (DAP), mean arterial pressure (MAP), and peripheral oxygen saturation (SpO2) were routinely monitored. A peripheral catheter was placed in all patients and started with 0.9% NaCl at 100 ml/h. After the patients were positioned, those who were given propofol sedation received 1 mg/kg IV bolus followed by propofol injection of 2.5 mg/kg/h. The patients in the propofol sedation group (Group I) were treated with a nasal cannula and 2–4 L/min of oxygen during the procedure. The patients in the sevoflurane anaesthesia group (Group II) were informed about the nasal mask application, and the masks were placed on their faces as demonstrated in Figure 1. They were told that they would continue to breathe and be under deep sedation. Then, propofol was given as an induction, at 1 mg/kg, and sevoflurane was administered with 50% oxygen at a 2% concentration. Sedation level control was determined as the achievement of a value equal to or greater than 4 RSS for all patients, followed by initiation of the procedure. In both groups, Group I with propofol infusion titration and Group II with an additional dose of propofol, the RSS values were between 4 and 5. The SpO2, HR, and blood pressure values were measured. Hypertension, tachycardia, and bradycardia were accepted as values greater or lower than 20% of baseline.

The adequacy of the patients’ sedation level was evaluated in 3 different steps using the 5-stage satisfaction scale. The degree of sedation and satisfaction of the gastroenterologist who performed the sphincterotomy were evaluated at the time of placement of the endoscope, when the sphincterotomy was performed and overall of procedure over 5 (1 for bad satisfaction and 5 for excellent satisfaction). The RSS values were recorded at these stages.

After the procedure, the patients were taken to the recovery room and kept there until the Aldrete score was 8 or over. Each patient’s eye-opening time and the duration of stay in the recovery room were noted. 

### 2.1. Statistical analysis

The SPSS 20.0 software package was used for statistical analysis (IBM Corp., Armonk, NY, USA). Mean, standard deviation, minimum and maximum values for continuous variables were employed to provide determinant statistical values; frequency and percentage values were calculated for categorical variables. Since our variables were normally distributed in the comparisons between groups (Kolmogorov–Smirnov test; P > 0.05), the independent-samples t-test and chi-square test were used to compare the categorical data. The results were evaluated at a 95% significance level. 

### 2.2. Ethical committee approval

The study protocol was carried out in accordance with the Helsinki Declaration as revised in 1989. Signed informed consent forms were obtained from all participants. Ethical approval was obtained from the Local Ethics Committee of Ankara Training and Research Hospital (ethical approval number: (0069 – 722)).

## 3. Results

The patients’ demographic characteristics are shown in Tables 1 and 2. There were 17 female and 14 male patients in Group I and 15 female and 15 male patients in Group II. In Group I, 9 patients (25.8%) with ASA 1, 19 (61.3%) with ASA 2 and 4 (12.9%) with ASA 3 criteria were anaesthetised. In Group II, 1 patient (3.3%) with ASA 1, 18 (60%) with ASA 2, and 11 (36.7%) with ASA 3 criteria were anaesthetised. There was no difference between the 2 groups in terms of demographic characteristics (Table 1).

**Table 1:** Demographic informations of patients.

			Frequency(n)	Percent(%)
Sex	Group 1	Female	17	54.8
		Male	14	45.2
		Total	31	100.0
	Group 2	Female	15	50.0
		Male	15
		50.0
Total	30	100.0
ASA criteria	Group 1	1	8	25.8
		2	19	61.3
		3	4	12.9
		Total	31	100.0
	Group 2	1	1	3.3
		2	18	60.0
		3	11	36.7
		Total	30	100.0

**Table 2:** (There is statistical difference between 2 groups at time of procedure and total propofol doses).

		n	Mean	Standard deviation	t; P
Time of procedure(min)	Group 1	31	20.48	10.17	-3,02; 0,004*
	Group 2	30	29.13	12.14	
Dose of propofol(mg)	Group 1	31	323.23	127.58	7.46; 0,001*
	Group 2	30	145.00	36.91	

*: Independent samples t-test; P < 0.05

The ERCP procedure was completed without interruption in all the patients. The procedure durations of the groups and propofol doses used are given in Table 2. The mean durations of treatment were 20.48 ± 10.17 min in Group I and 29.13 ± 12.14 min in Group II. There was a statistically significant difference between the 2 groups in terms of processing times (P= 0.004). The mean propofol dose was 323.23 mg ± 127.58 mg in Group I, while it was 145 mg ± 36.91 mg in Group II. There was a statistically significant difference between the propofol dose amounts of the 2 groups (P= 0.001). 

There was no statistically significant difference between the RSS values at 10, 20, and 30 min in Groups I and II (P > 0.05). (Table 3) There was no statistically significant difference between the placement time satisfaction score values in Groups I and II (P > 0.05).

**Table 3:** (There is no significant difference between 2 groups at median Aldrete scores at 10th min, 20th min, and 30th min).

			10th min Ramsay	20th min Ramsay	30th min Ramsay	40th min Ramsay
Group 1	n	Valid	26	14	6	1
		Missing	5	17	25	30
	Median		6.00	6.00	5.50	6.00
	Minimum		5	5	5	6
	Maximum		6	6	6	6
Group 2	n	Valid	30	23	16	7
		Missing	0	7	14	23
	Median		6.00	6.00	6.00	6.00
	Minimum		4	4	4	5
	Maximum		6	6	6	6

*Mann–Whitney U test; P > 0.05

The mean sphincterotomy satisfaction score of Group I was 4.32 ± 0.83, while that of Group II was 3.67 ± 1.52, and there was a statistically significant difference between the groups (P= 0.043). However, the overall satisfaction scores were found to be similar in the 2 groups (P > 0.05; Table 4).

**Table 4:** (There statistically significant difference between two groups at eye opening time, time of recovery and sphincterotomy satisfaction)

		n	Mean	Standard Deviation	t;p
First enterance stage of endoscope Satisfaction	Group 1	31	4,39	,88	0,61; 0,543
	Group 2	30	4,27	,64	
Sphincterotomy Satisfaction	Group 1	31	4,32	,83	2,09; 0,043*
	Group 2	30	3,67	1,52	
Total Satisfaction	Group 1	31	4,2	,8	0,056
	Group 2	30	4,1	,81	
Time of Recovery	Group 1	31	8,03	3,34	-3,99; 0,001*
	Group 2	30	12,80	5,71	
Eye opening time	Group 1	31	4,74	3,38	-2,87; 0,006*
	Group 2	30	8,67	6,72	

*Independent Samples t test p<0,05

The starting oxygen saturation levels were statistically different (P > 0.05; Table 5). The mean saturation level of Group I was 97.52, while that of Group II was 96.03 (P = 0.006). However, there was no significant difference between the 10-, 20- and 30-min levels. The median Aldrete scores were similar, at 10 (min. 9, max. 10 in both groups) (Table 6).

**Table 5:** There is a statistical difference at Starting SpO_2_, but there is no statistical difference with other values.

		n	Mean	Standard deviation	t; P
Preoperative SpO_2_	Group 1	31	97.52	1.57	2.86; 0.006 *
	Group 2	30	96.03	2.39		10th min SpO_2_	Group 1	26	97.73	2.24	-1.11; 0.274
Group 2	30	98.30	1.47								
20th min SpO_2_	Group 1	14	98.43	1.28	0.55; 0.588
	Group 2	22	98.68	1.39	
30th min SpO_2_	Group 1	6	99.00	.63	0.33; 0.749
	Group 2	15	99.13	.92	

*:Independent samples t-test (P < 0.05)

**Table 6:** (There is no statistical difference between 2 groups at median values of Aldrete Score).

			Aldrete
Group 1	n	Valid	31
		Missing	0
	Median		10.00
	Minimum		9
	Maximum		10
Group 2	n	Valid	30
		Missing	0
	Median		10.00
	Minimum		9
	Maximum		10

Mann–Whitney U test; P > 0.05

**Figure 2:**
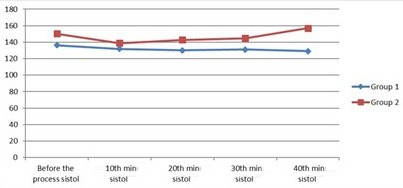
Systolic pressure values of 2 groups (procedure and recovery).

**Figure 3:**
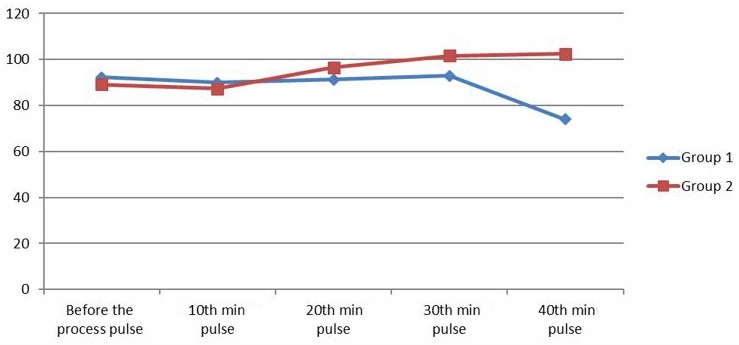
Pulse values of 2 groups (procedure and recovery).

The mean duration of Group I from the end of the procedure to the patient opening his/her eyes was 4.74 ± 3.38 min, and the mean duration of Group II was measured as 8.67 ± 6.72 min. There was a statistically significant difference between the 2 groups (P = 0.006). During the procedure, the blood pressure, arterial pressure, HR, and oxygen saturation were similar in both groups (Figures 1, 2, 3, Table 5).

## 4. Conclusion

In this study, sedation with the nasal mask sevoflurane and oxygen combination in adult patients undergoing ERCP resulted in a similar outcome hemodynamic parameters, total operator satisfaction, and Aldrete scores. At propofol group, recovery duration and eye-opening time statistically shorter. The ERCP procedure has become increasingly important in the management and treatment of biliary and pancreatic diseases. It has been reported that sedation is important enough to be successful in this process, and intolerance to sedation directly affects ERCP failure. While the sedation depth can be adjusted, some scales or devices, such as the BIS, that are connected to the numerical evaluation principle of brainwaves similar to EEG waves can be used by anaesthetists [5]. 

Anaesthetic approaches range from conscious sedation to deep sedation in procedures where advanced technology like ERCP and endoscopic ultrasonography (USG) are used worldwide [6]. Propofol, which is an intravenous agent, is preferred for sedation due to its fast and soft induction, good hypnotic properties, short duration of action and fast and comfortable awakening. Because of these properties, with a rate of 42.3%, it became the preferred agent for ERCP procedures in the United States in 2011 [7,8]. In addition, propofol is known to cause hypotension, with a dose-dependent temporary hypoxia of 3%–7% and 4%–7% [9]. In one study, it was stated that the hemodynamic comparison of propofol and etomidate for ERCP showed a greater prominence of propofol-induced hypotension and tachycardia. It was also mentioned that, especially in patients with ASA 3 and those over 50 years of age, for avoiding severe hypotension, an alternative drug to propofol should be used [10]. Lim et al. [11] reported that premedication of patients with midazolam before propofol induction reduced the incidence of hypotension.

In terms of the reliability of sedation procedures, the airway, respiration, oxygenation and end-tidal carbon dioxide monitoring are extremely important. For this, the protection of spontaneous breathing is recommended [12]. In addition, oxygen support should be given regardless of the sedation level. If general anaesthesia is not applied in ERCP procedures, nasal oxygen support should be given unconditionally because hypoxia may develop. There are too many oxygen application techniques in this regard; however, because of the difficulties in heating and humidification, oxygen is generally used at a low current (up to 15 L).

Different positions of the patient, such as supine, side and prone, may make breathing control difficult. In our centre, patients were treated in a lateral position using nasal oxygen or a nasal mask with sevoflurane and oxygen. Sevoflurane is an anaesthetic agent that provides rapid induction and rapid stimulation due to its low blood-gas partition coefficient, lack of irritant odour, ability to increase secretion and lack of generation of airway spasm. In addition, adverse cardiovascular effects and respiratory depression are also lower compared with when IV agents are employed. The nasal mask applied in our study has been used in dentistry procedures in the literature to give nitrogen protoxide only for paediatric patients [13]. From this point of view, in our study, sufficient ERCP quality was achieved via the application of nasal 1 MAC sevoflurane after IV induction. Although the initial oxygen saturation of the patients in this group was lower than that of in the propofol group, they were not hypoxic during the procedure. This can be considered as an advantage, especially for patients with respiratory distress. Volatile anaesthetics caused the relaxation of smooth airway muscle by several mechanisms that reduce intracellular free calcium in in vitro studies [14]. Clinically, sevoflurane and other volatile anaesthetics are known to be potent bronchodilators, and they have even been used as potentially lifesaving therapeutic agents in the treatment of status asthmatics for several years [15].

Use of higher MAC sevoflurane was not preferred during the procedure. Instead, additional propofol was applied to the patient according to the RSS. At this point, we found this limitation to be appropriate, since the patient would be spreading sevoflurane out of his/her mouth. In the situations where open systems are used, anaesthetic gas is released to the environment when mask ventilation is present. Although there is a gas waste and ventilation system in the environment, the number of patients taken daily is restricted to 2 to minimise this amount. In addition, it is assured that the nasal mask is fully seated and the gas concentration is limited.

It is reported in the literature that sevoflurane is used safely in intubated patients, especially in endoscopic interventions in children. In one study, tracheal intubation was not preferred for short-term endoscopic procedures in children, and oxygen + nitrous oxide + sevoflurane was applied with a face mask [16]. In this application, it was reported that the patients were not intubated, but the endotracheal tube was placed in the mouth and insufflation was performed through this. In the study, when the transaction/operation comfort of the patients (colonoscopy) and the rate of recovery was compared with the propofol group and the fentanyl-ketamine-midazolam group, it was determined that equal treatment comfort was achieved in the sevoflurane group, and the rate of recovery and duration of discharge were shorter than in the 2 groups.

In another study by Gomes et al. [13], an oral midazolam + ketamine + nasal mask with oxygen group and nasal mask sedation concentration of sevoflurane + oxygen applied group were examined in children’s oral dental treatment. In this pilot study, haemodynamic parameters (HR, oxygen saturation) were found to be similar during the procedure and at post – anaesthesia recovery. In children receiving sevoflurane with a nasal mask, crying and head movement were seen less under local anaesthesia compared with the other group. The authors determined that sevoflurane had control over injection pain and better control of behaviour than was evident in the other group. Children who received sevoflurane were observed to cry, but they moved less than those who received oxygen. In this study, the authors could not say that they obtained obvious superiority with sevoflurane. They also stated that they did not follow any increasing side effects.

Oxygen application alone with a nasal mask is important for airway safety in every way. As an alternative, the application of moistened, high-flow nasal oxygen was also performed by Schuman et al. [12]. In this retrospective study, high flow nasal oxygen (HFNO), mainly used in hypoxemic respiratory failure, was used to reduce the need for ERCP and endoscopic ultrasound (EUS) general anaesthesia in adults. The researchers reported a significant decrease in general anaesthesia applications in the 3-month period when HFNO was used and a significant increase in oxygen saturation according to the cases in the last 3 months. The importance of oxygen support in patients with similar ASA levels and the rate of passing to general anaesthesia in patients were indicated. In the literature, the upper gastrointestinal endoscopic procedures indicated 60% cardiopulmonary events during sedation and anaesthesia [17]. The prolongation of the procedure poses a risk, especially for hypoxic periods [18]. Transition to general anaesthesia is not preferred for the patient in short-term procedures. For these reasons, it is necessary to support the airway strongly, especially for the procedures requiring deep sedation.

The modified Aldrete score used in the recovery period of the patients is a scoring system that helps to determine whether patients should be sent to post anaesthetic chambers, both after general anaesthesia and after sedation [19]. The duration of eye- opening and length of stay in the propofol group found to be shorter than the sevoflurane group. However, the total duration of staying in recovery was as short as 12 min and it was not evaluated as a difference that would force clinicians in practice. In this duration, Aldrete scores reached the same level in both groups. 

As our study’s result, propofol infusion is preferred in ERCP procedures due to its short recovery duration and adequate sedation levels. It is concluded that sevoflurane + oxygen sedation with nasal mask in ERCP cases can be chosen as a “patient-specific anaesthesia”, especially for airway support and safety treating hypoxemic patients.

## Acknowledgments/Disclaimers

The authors appreciate the contributions and statistical assistance made by Murat Mutlu. There is no external funding for this manuscript.

## Informed Consent

The study protocol was carried out in accordance with the Helsinki Declaration as revised in 1989. Signed informed consent forms were obtained from all participants. Ethical approval was obtained from the Local Ethics Committee of Ankara Training and Research Hospital (ethical approval number: (0069 – 722)).

## References

[ref0] (2014). Safety in the Gastrointestinal Endoscopy Unit Task Force. Gastrointestinal Endoscopy.

[ref1] (2018). Complications caused by nitrous oxide in dental sedation. Journal of Dental Anesthesia and Pain Medicine.

[ref2] (2013). Application of dual mask for postoperative respiratory support in obstructive sleep apnea patient. Case Reports in Anesthesiology.

[ref3] (2002). Evaluation of endoscopic retrograde cholangiopancreatography under conscious sedation and general anesthesia. Endoscopy.

[ref4] (2006). Success of Repeat ERCP Following Initial Therapeutic Failure. Gastrointestinal Endoscopy.

[ref5] (2014). Propofol use in endoscopic retrograde cholangiopancreatography and endoscopic ultrasound. World Journal of Gastroenterology.

[ref6] (2012). Utilization of anesthesia services during outpatient endoscopies and colonoscopies and associated spending in 2003-2009. Journal of American Medical Association.

[ref7] (2017). Anesthesia service use during outpatient gastroenterology procedures continued to increase from 2010 to 2013 and potentially discretionary spending remained high. American Journal of Gastroenterology.

[ref8] (2008). Standards of Practice Committee of the American Society for Gastrointestinal Endoscopy. Gastrointestinal Endoscopy.

[ref9] (2009). Baroreflex sensitivity is impaired in patients with obstructive jaundice. Anesthesiology.

[ref10] (2012). The cardiovascular effects of midazolam co-induction to propofol for induction in aged patients. Korean Journal of Anesthesiology.

[ref11] (2016). High-flow nasal oxygen availability for sedation decreases the use of general anesthesia during endoscopic retrograde cholangiopancreatography and endoscopic ultrasound. World Journal of Gastroenterology.

[ref12] (2017). Does sevoflurane add to outpatient procedural sedation in children? A randomised clinical trial. BioMed Central Pediatrics.

[ref13] (2003). Cellular mechanisms of airway smooth muscle relaxant effects of anesthetic agents. Journal of Anesthesia.

[ref14] (2015). Volatile anesthetics and the treatment of severe bronchospasm: a concept of targeted delivery. Drug Discovery Today Disease Models.

[ref15] (2000). Deep sedation with inhaled sevoflurane for pediatric outpatient gastrointestinal endoscopy. Journal of Pediatric Gastroenterology and Nutrition.

[ref16] (2007). A national study of cardiopulmonary unplanned events after GI endoscopy. Gastrointestinal Endoscopy.

[ref17] (1990). Effect of intranasal oxygen on hypoxia and tachycardia during endoscopic cholangiopancreatography. British Medical Journal.

